# Evolutionary Dynamics of the Mitochondrial Genome in the Evaniomorpha (Hymenoptera)—A Group with an Intermediate Rate of Gene Rearrangement

**DOI:** 10.1093/gbe/evu145

**Published:** 2014-07-03

**Authors:** Meng Mao, Tracey Gibson, Mark Dowton

**Affiliations:** Centre for Medical Bioscience, School of Biological Sciences, University of Wollongong, New South Wales, Australia

**Keywords:** mitochondrial genome, Evaniomorpha, Aculeata, phylogeny, gene rearrangement

## Abstract

We determined the complete mitochondrial (mt) genomes of three evaniomorph species, *Ceraphron* sp. (Ceraphronoidea), *Gasteruption* sp. (Evanioidea), and *Orthogonalys pulchella* (Trigonalyoidea) as well as the nearly complete mt genome from another evaniomorph species, *Megalyra* sp. (Megalyroidea). Each of them possesses dramatic gene rearrangements, including protein-coding or rRNA genes. Gene inversions were identified in all of these mt genomes; for example, the two rRNA genes have inverted and moved into the *nad2-cox1* junction in the *Megalyra* sp. mt genome. In addition, we found two copies of a 10-bp complementary repeat at the beginning of *rrnS* and at the end of *trnL_2_* in the *Gasteruption* sp. mt genome, consistent with recombination as the possible mechanism for gene inversion and long-range movement. Although each of the genomes contains a number of repeats of varying size, there was no consistent association of the size or number of repeats with the extent or type of gene rearrangement. The breakpoint distance analysis showed the Evaniomorpha has an intermediate rate of gene rearrangement. Sequence-based phylogenetic analyses of 13 protein-coding and 2 rRNA genes in 22 hymenopteran taxa recovered a paraphyletic Evaniomorpha with the Aculeata nested within it. Within the Evaniomorpha, our analyses confirmed the Trigonalyoidea + Megalyroidea as the sister group to the Aculeata and recovered a novel clade, Ceraphronoidea + Evanioidea. In contrast to previous hymenopteran phylogenetic studies, the internal relationships of the Evaniomorpha were highly supported and robust to the variation of alignment approach and phylogenetic inference approach.

## Introduction

Knowledge of the forces that shape the organization of the animal mitochondrial (mt) genome has suffered from a lack of suitable model systems. This is because the rate of gene rearrangement in most animal mt genomes is generally extremely slow; for example, most vertebrates have identically arranged mt genomes (Boore 1999). The ideal model system would provide multiple gene rearrangements for comparison (to identify common themes), but in a lineage that is not rearranging so frequently that hidden and convergent rearrangements obscure the interpretation of evolutionary events. In the insects, a number of higher level lineages have been identified with accelerated rates of mt gene rearrangement—the Hymenoptera (Dowton and Austin 1999) and the hemipteroid orders (Shao et al. 2001) are two examples. Within these rapidly rearranging lineages, taxonomic sampling at lower levels may be all that is required to identify good model groups; that is, ones in which the evolutionary trajectory of genome reorganization is straightforward to interpret. In this study, we investigated whether one group of the Hymenoptera (the Evaniomorpha) might provide such a model system.

The Hymenoptera (sawflies, wasps, ants, and bees) is one of the most important components of insect diversity. As the third largest insect order, it is estimated to contain more than 140,000 extant species, placed into two traditional suborders (Symphyta and Apocrita) (Huber 2009). The Apocrita has long been considered as a natural group and comprises more than 90% of the Hymenoptera (Sharkey 2007; Huber 2009). Many apocritan species play valuable roles in biological control, ecosystem, and production of commercial products (LaSalle and Gauld 1993).

**Fig. 1.—:**
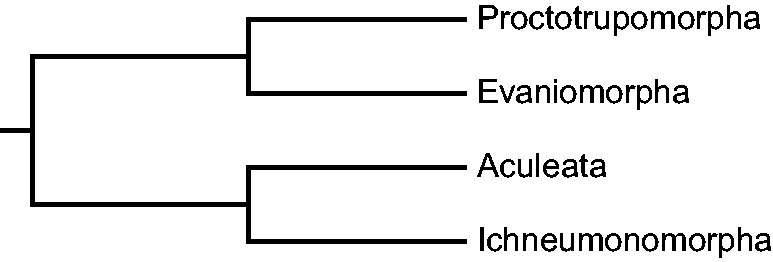
Simplified interpretation of the phylogenetic relationships among the major lineages within the Apocrita from Rasnitsyn (1998).

Traditionally, the Apocrita is subdivided into two groups, the Aculeata (stinging wasps) and the Parasitica (parasitoid wasps), with the Aculeata now widely accepted as being derived from within the Parasitica (Sharkey 2007). The reconstruction of a robust phylogeny for the Apocrita has long been of great interest to hymenopteran systematists (reviewed by Sharkey [2007]). In 1988, Rasnitsyn proposed a fully resolved phylogenetic hypothesis of higher level hymenopteran relationships based on morphological and fossil evidence (Rasnitsyn 1988). Despite the use of noncladistic methodology, Rasnitsyn’s research remains influential and sets a stage for current research of hymenopteran phylogeny. In his hypothesis, the Apocrita was divided into four lineages, the Ichneumonomorpha, the Vespomorpha (Aculeata), the Proctotrupomorpha, and the Evaniomorpha (fig. 1). The Ichneumonomorpha and Aculeata have long been recovered as natural groups (Dowton et al. 1997; Dowton and Austin 2001; Castro and Dowton 2006; Rasnitsyn and Zhang 2010; Vilhelmsen et al. 2010; Heraty et al. 2011; Sharkey et al. 2011; Klopfstein et al. 2013), and the monophyly of the Proctotrupomorpha has been supported with several comprehensive studies (Castro and Dowton 2006; Heraty et al. 2011; Sharkey et al. 2011; Klopfstein et al. 2013). However, the monophyly of the Evaniomorpha (including the Ceraphronoidea, Evanioidea, Megalyroidea, Stephanoidea, and Trigonalyoidea) remains unclear from both morphological and molecular analyses. In morphological analyses, a subsequent numerical cladistic analysis of Rasnitsyn’s (1988) data failed to retrieve the Evaniomorpha (Ronquist et al. 1999). Gibson (1999) suspected that the mesocoxal articulatory structure, which supported the monophyly of the Evaniomorpha according to Rasnitsyn (1988), was a retained symplesiomorphy rather than a synapomorphy (Gibson 1999). Moreover, Rasnitsyn himself proposed that the Evaniomorpha was not monophyletic and divided it into three lineages (Rasnitsyn and Zhang 2010). Most molecular analyses recovered the Evaniomorpha as paraphyletic, with the Aculeata nested within the Evaniomorpha. Further, the sister group of the Aculeata varied among different analyses (Castro and Dowton 2006; Heraty et al. 2011; Sharkey et al. 2011; Klopfstein et al. 2013). Within the Evaniomorpha, there is little consensus on the phylogenetic relationships among the superfamilies due to a lack of reliable morphological characters and the limited number of molecular markers.

Prior to this study, there were only four evaniomorph mt genomes available in GenBank, representing three superfamilies (*Evania appendigaster* [Evanioidea: Evaniidae]; *Pristaulacus compressus* [Evanioidea: Aulacidae]; *Schlettererius cinctipes* [Stephanoidea: Stephanidae]; and *Conostigmus* sp. [Ceraphronoidea: Megaspilidae]) (Dowton, Cameron, Austin, et al. 2009; Wei, Tang, et al. 2010; Wei et al. 2013; Mao et al. 2014). Each of these mt genomes displayed a relatively small number of changes to their mt genome organization, when compared with the ancestral pancrustacean/hymenopteran organization, and these rearrangements involved both tRNA genes and (to a lesser extent) protein-coding genes. *Schlettererius* had evidence of two tRNA gene rearrangements (Dowton, Cameron, Dowavic, et al. 2009), *Pristaulacus* had evidence of one protein-coding gene (*nad1*) rearrangement and two tRNA gene rearrangements (Wei et al. 2013), *Evania* had evidence of four tRNA gene rearrangements (Wei, Tang, et al. 2010), whereas *Conostigmus* had 2 protein-coding genes rearranged (*cox1*, *nad2*) and 10 tRNA genes rearranged (Mao et al. 2014). Thus, this group represents a good candidate model system in which to investigate the dynamics of mt genome rearrangement, as individual rearrangements might be inferred through denser taxonomic sampling. For this reason, in this study we present four new mt genomes for representatives of four evaniomorph superfamilies, *Megalyra* sp. (Megalyroidea: Megalyridae), *Orthogonalys pulchella* (Trigonalyoidea: Trigonalyidae), *Ceraphron* sp. (Ceraphronoidea: Ceraphronidae) and *Gasteruption* sp. (Evanioidea: Gasteruptiidae). We also use these entire mt genomes to further investigate relationships among the Evaniomorpha and Aculeata. Because of the likely paraphyletic nature of the Evaniomorpha, we include the Aculeata to examine the phylogeny within a natural group.

## Materials and Methods

### DNA Extraction

The collection details for each study species are listed in table 1. Genomic DNA was extracted from 100% ethanol preserved specimens using the “salting out” protocol (Aljanabi and Martinez 1997). The DNA was resuspended in 100 μl of fresh TE solution (1 mM Tris–HCl, 0.1 mM ethylenediaminetetraacetic acid [pH 8]) and stored at 4° C.

**Table 1:** Species Sequenced in This Study, Collection Data, and GenBank Accession Numbers

Species	Library Reference	Family	Superfamily	Collection Locality	Accession Number
*Ceraphron* sp.	M247	Ceraphronidae	Ceraphronoidea	CA	KJ570858
*Gasteruption* sp.	M19	Gasteruptionidae	Evanioidea	Auburn, South Australia	KJ619460
*Megalyra* sp.	M272	Megalyridae	Megalyroidea	Wistow, South Australia	KJ577600
*Orthogonalys pulchella*	M16	Trigonalyidae	Trigonalyoidea	Clarke County, WA	KJ619461

### Mt Genome Amplification, Sequencing, and Annotation

Short gene fragments were amplified and sequenced using a range of universal insect mt primers (Simon et al. 1994, 2006) and primers that had been previously designed from consensus hymenopteran mt sequences. Using the sequence information obtained, taxon-specific primers were designed for each sample to amplify the remaining regions. Polymerase chain reaction and sequencing reactions were conducted as previously described (Mao et al. 2014). The sequences of both strands were determined for the entire mt genomes of *Ceraphron* sp., *Gasteruption* sp., and *O. pulchella*, In addition, we sequenced both strands for all coding regions and most of the A+T-rich region of *Megalyra* sp. but failed to get the full double-stranded sequence for the entire A+T-rich region due to an array of 50-bp tandem repeats. This array contained 20 repeats, and as a result, no unique internal sequencing primers could be designed.

Raw sequences were assembled into contigs in ChromasPro Ver 1.33 (Technelysium Ltd., Tewantin, Australia). tRNA genes were identified using tRNA-scan SE 1.21 (lowelab.ucsc.edu/tRNAscan-SE/, last accessed June 30, 2014) (Lowe and Eddy 1997) and ARWEN 1.2 (http://130.235.46.10/ARWEN/, last accessed June 30, 2014) (Laslett and Canbäck 2008). ORFinder (www.ncbi.nlm.nih.gov/gorf/gorf.html, last accessed June 30, 2014) was used to identify protein-coding genes, specifying the invertebrate mt genetic code. The start and stop codons of some genes were corrected according to the boundaries of tRNA genes and through alignment with other hymenopteran mt sequences. rRNA genes were identified using BLASTN, and the ends of genes were assumed to extend to the boundaries of the neighboring tRNA or protein-coding genes. However, the boundary between *rrnL* and *rrnS* was difficult to define in *Megalyra* sp., as there is no gene between them. In this case, we determined this boundary through sequence comparison with published hymenopteran mt rRNA sequences.

### Nucleotide Composition, Gene Rearrangement, and Repeat Analyses

The A+T content was determined for the major strand of each of the four mt genomes by MEGA5 (Tamura et al. 2011). To investigate whether gene inversions influenced the nucleotide compositional biases, we also measured the AT and GC skews for some protein-coding genes (*nad2*, *cox1*, *nad6,* and *cob*) and two rRNA genes of 8 evaniomorph taxa and 12 aculeate taxa. These genes were inverted in at least one of the four new mt genomes (see below). The formulae used were AT-skew = (A − T)/(A + T) and GC-skew = (G − C)/(G + C) (Perna and Kocher, 1995).

Gene rearrangement analyses were conducted with CREx via the freely available CREx web server (http://pacosy.informatik.uni-leipzig.de/crex, last accessed June 30, 2014) (Bernt et al. 2007). We compared gene orders using a dissimilarity measurement—number of breakpoints and visually inspected the output diagram to identify shared, derived gene rearrangements.

Direct and inverted repeats longer than 12 bp were identified for the A+T-rich region and the entire mt genome of each evaniomorph taxa using UGENE v.1.13.1 (Okonechnikov et al. 2012). We calculated the number of different size repeats around the breakpoints and in the A+T-rich region to investigate whether any particular size of repeat facilitate gene rearrangement.

### Sequence Alignment and Phylogenetic Analysis

A total of 24 taxa were analyzed in this study, including 8 evaniomorph taxa, 12 aculeate taxa, and 4 symphytan taxa. Nucleotide sequences for each of the 13 protein-coding genes and the 2 rRNA genes were imported into separate files using MEGA5 and aligned using Muscle (Edgar 2004) or MAFFT (Katoh et al. 2005). For the protein-coding genes (excluding the stop codons), an amino acid alignment was generated first for each gene in Muscle as implemented within MEGA5 or MAFFT at the freely available TranslatorX server (http://translatorx.co.uk/, last accessed June 30, 2014) (Abascal et al. 2010). A nucleotide alignment was then inferred from the amino acid alignment. The alignment parameters for all genes in Muscle were the default settings, which have been specified in a previous study (Mao et al. 2012). For the MAFFT alignment of the rRNA genes, we used the G-INS-i algorithm as implemented in the MAFFT web server (http://mafft.cbrc.jp/alignment/server/, last accessed June 30, 2014) (Katoh et al. 2005). MAFFT (G-INS-i) has been shown to be more accurate than other programs (Golubchik et al. 2007). No regions were excluded from the rRNA alignments. Individual gene alignments were concatenated prior to phylogenetic analysis.

The best partitioning schemes and corresponding nucleotide substitution models were determined with PartitionFinder version 1.0.1 (Lanfear et al. 2012) using the Bayesian Information Criterion and a heuristic search algorithm. A total of 41 data blocks were predefined (3 codon positions of 13 protein-coding genes + 2 rRNA genes). Maximum likelihood and Bayesian approaches were employed to infer phylogenetic trees. Maximum likelihood analyses were conducted with RAxML via the available RAxML BlackBox server (http://embnet.vital-it.ch/raxml-bb/, last accessed June 30, 2014) (Stamatakis et al. 2008). The GAMMA model of rate heterogeneity was employed for all partitions. The “Maximum likelihood search” and “Estimate proportion of invariable sites” boxes were selected, with a total of 100 bootstrap replicates performed. Bayesian analyses were conducted with MrBayes v. 3.2.2 (Ronquist et al. 2012) via the online CIPRES Science gateway portal (Miller et al. 2010). The Markov chain Monte Carlo process was set, so that four chains (three heated and one cold) ran simultaneously. Four separate runs using unlinked partitions (unlink statefreq = all; unlink revmat = all; unlink shape = all; unlink pinvar = all; prset applyto = all; ratepr = variable) for a total of 1,000,000 generations were performed, with sampling every 100 generations. Stationarity for each run was assessed by importing the parameter files into Tracer v. 1.5 (Rambaut and Drummond 2009).

### Rogue Taxon Identification

“Rogue” taxa, defined as those that have an unstable position in topological trees (Wilkinson 1996), can obscure relationships that are consistently recovered during bootstrap and Bayesian analyses (Aberer et al. 2013). To reveal those relationships that were consistently recovered by our analyses, we conducted rogue taxon filtering using the approach implemented in RogueNaRok (http://rnr.h-its.org/rnr, last accessed June 30, 2014) (Aberer et al. 2013). The RAxML bootstrap tree sets and Bayesian tree sets (excluding burn-in) were employed to perform these analyses.

## Results and Discussion

### General Features of mt Genomes

**Fig. 2.—:**
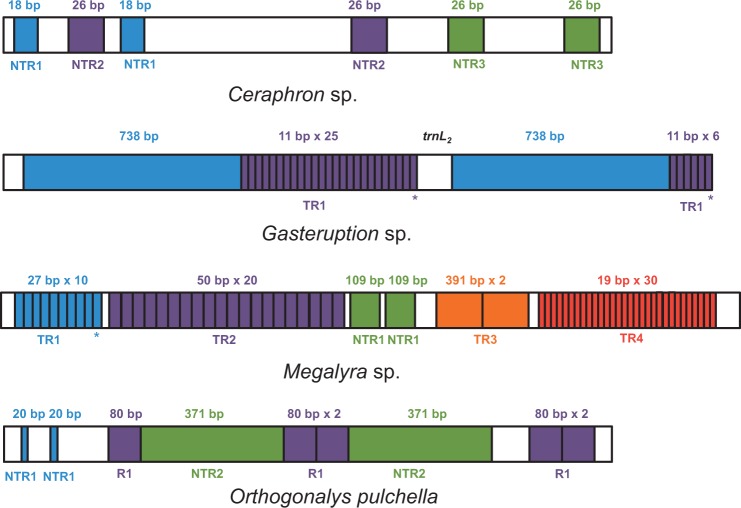
Structure of the A+T-rich region in the *Ceraphron* sp., *Gasteruption* sp., *Megalyra* sp., and *Orthogonalys pulchella* mt genomes. TR1-N and NTR1-N indicate the distinct tandem repeats and nontandem repeats in each genome, respectively. The 80-bp repeat in the *O. pulchella* mt genome, which was difficult to classify as either tandem or nontandem, is labeled as R1. The length and copy number of each repeat are indicated above each repeat. The small repeats that nest within the large repeats are not shown. The width of different boxes is not proportional but indicative of the size of a repeat within a particular genome. Different repeats in each mt genome are indicated with different colors. Boxes with asterisks underneath represent a partial copy of a repeat.

Three complete and one nearly complete mt genomes were sequenced for this study: *Ceraphron* sp. (14,947 bp), *Gasteruption* sp. (17,884 bp), *O. pulchella* (17,277 bp), and *Megalyra* sp. (18,996 bp). For *Megalyra* sp., we were able to estimate the size of the genome with a reasonable degree of precision, because imperfect copies exist in the array of 50-bp tandem repeats, for which we failed to get the full double-stranded sequence. Each genome contains all of the 37 genes commonly found in animal mt genomes (Boore 1999). In addition, a second copy of *trnE* was identified in the *O. pulchella* mt genome. As the shortest hymenopteran complete mt genome to date, the *Ceraphron* sp. mt genome is extremely compact with a total of only 105 bp of short noncoding regions (excluding the A+T-rich region). The short noncoding regions in *Megalyra* sp., *Gasteruption* sp., and *O. pulchella* are 345-bp, 909-bp, and 919-bp long, respectively. The four mt genomes have a similar A+T content, ranging from 80% (*Ceraphron* sp.) to 83.8% (*O. pulchella*). The AT and GC skew analysis showed that gene inversions had no influence on nucleotide compositional biases (data not shown). Three of the conventional start codons (ATA, ATG, or ATT) could be assigned to all of the protein-coding genes. Most tRNA genes have a typical cloverleaf structure. The exceptions are *trnS_1_* in all four species, *trnS_2_* in *Ceraphron* sp. and *trnR* in *Ceraphron* sp. and *Gasteruption* sp. All of these tRNA genes lack the D-stem pairings in the dihydrouridine (DHU)-arm. The missing D-stem has been commonly noted for the *trnS_1_* gene in insects (Sheffield et al. 2008; Mao et al. 2012; Yang et al. 2013), while to our knowledge, it has not been previously reported in *trnS_2_* in insect mt genomes. The missing D-stem in *trnR* is likely a common feature of hymenopteran mt genomes. In addition to the two mt genomes reported here, it was also found in another two evaniomorph taxa (*Conostigmus* sp. and *Schlettererius cinctipes*) (Dowton, Cameron, Austin, et al. 2009; Mao et al. 2014) and in two sceliond mt genomes (*Ceratobaeus* sp. and *Idris* sp.) sequenced in our laboratory (Mao and Dowton 2014). Furthermore, the 3′-end of the aminoacyl acceptor stem of *trnR* overlaps with the downstream gene in *Ceraphron* sp., *Megalyra* sp., and *O. pulchella*. The truncated aminoacyl acceptor stem is probably restored to conventional structure by extensive tRNA editing (Dowton and Austin 1999; Lavrov et al. 2000; Masta and Boore 2004; Segovia et al. 2011). The considerable difference in genome size among the four evaniomorph taxa is mainly due to variation in the size of the A+T-rich region, which ranges from 692 bp (*Ceraphron* sp.) to 3,871 bp (*Megalyra* sp.). One or more series of tandem repeats could be found in the *Megalyra* sp., *O. pulchella*, and *Gasteruption* sp. mt genomes, whereas only three short nontandem repeats are present in the *Ceraphron* sp. mt genome (fig. 2 and supplementary fig. S1*A*, Supplementary Material online). Most notably, two copies of the A+T-rich region exist in the *Gasteruption* sp. mt genome, which are separated by *trnL_2_*. The second copy is 77.7% of the length of the first copy and the length difference is mainly due to the variation in repeat number of an 11-bp microsatellite sequence (supplementary fig. S1*B*, Supplementary Material online). Similarly, the major portion of the A+T-rich region in the *O. pulchella* mt genome is also duplicated (supplementary fig. S1*C*, Supplementary Material online). The presence of duplicated A+T-rich regions (control regions) has been reported in a diverse range of taxa, such as mantellid frogs (Kurabayashi et al. 2008), *Amazona* parrots (Eberhard et al. 2001), and Australasian *Ixodes* ticks (Shao et al. 2005). It is proposed that the presence of two control regions may be advantageous and be maintained either through stabilizing selection or through gene conversion (Eberhard et al. 2001).

### Genome Organizations

**Fig. 3.—:**
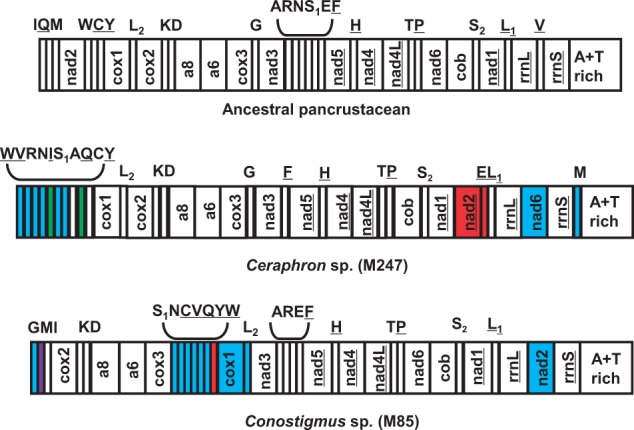
mt genome organization of two taxa from the Ceraphronoidea, *Ceraphron* sp. (present study) and *Conostigmus* sp*.* (Mao et al. 2014), compared with the ancestral pancrustacean/hymenopteran mt genome organization. tRNA genes are indicated by single-letter amino acid codes, *L_1_*, *L_2_*, *S_1_,* and *S_2_* denote *trnL^CUN^*, *trnL^UUR^*, *trnS^AGN^,* and *trnS^UCN^*, respectively. Genes are transcribed from left to right except those indicated by underlining. Different types of gene movements, relative to the ancestral organization, are indicated with different colors: Blue represents long-distance translocation; red represents long-distance translocation and inversion; purple represents local translocation; green represents local inversion.

**Fig. 4.—:**
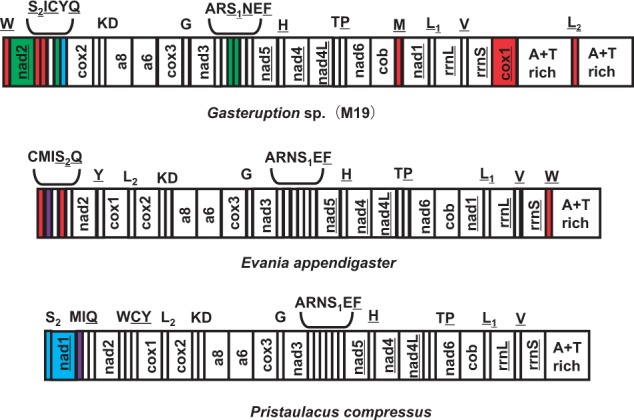
mt genome organization of three taxa from the Evanioidea: *Gasteruption* sp. (present study), *Evania appendigaster* (Wei, Tang, et al. 2010), and *Pristaulacus compressus* (Wei et al. 2013). Genes are transcribed from left to right except those indicated by underlining. Different types of gene movements, relative to the ancestral organization, are indicated with different colors: Blue represents long-distance translocation; red represents long-distance translocation and inversion; purple represents local translocation; green represents local inversion.

**Fig. 5.—:**
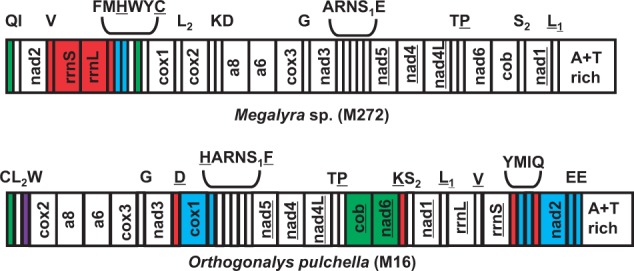
mt genome organization of *Megalyra* sp. (Megalyroidea) (present study) and *Orthogonalys pulchella* (Trigonalyoidea) (present study). The sister relationship of these two superfamilies is supported by present study and some other molecular studies. Genes are transcribed from left to right except those indicated by underlining. Different types of gene movements, relative to the ancestral organization, are indicated with different colors: Blue represents long-distance translocation; red represents long-distance translocation and inversion; purple represents local translocation; green represents local inversion.

The four mt genome organizations are shown in figures 3–5. All of them possess dramatic gene rearrangements when compared with the ancestral pancrustacean mt genome organization (Cook 2005), which is also thought to represent the ancestral organization of the hymenopteran mt genome (Mao et al. 2012). Remarkably, each of the newly sequenced mt genomes has protein-coding or rRNA gene rearrangement(s), a feature which has not been commonly reported in the Hymenoptera (only 8 of the 38 available mt genomes [this number excludes congenerics] have protein-coding or rRNA gene rearrangements) (Castro et al. 2006; Oliveira et al. 2008; Dowton, Cameron, Dowavic, et al. 2009; Wei, Shi, et al. 2010; Xiao et al. 2011; Wei et al. 2013; Mao et al. 2014).

### The mt Genome of *Ceraphron* sp.

In *Ceraphron* sp., two protein coding genes (*nad2* and *nad6*) and 10 tRNA genes have rearranged relative to their ancestral positions. The tRNA gene rearrangements mainly occur at the *nad3-nad5* junction and around the A+T-rich region. Four tRNA genes (*trnA*, *trnR*, *trnN,* and *trnS_1_*) have moved from the *nad3-nad5* junction to the tRNA gene cluster downstream of the A+T-rich region. *trnE* has also moved out of the *nad3-nad5* junction, and we propose that it might be involved in two gene rearrangement events. First, it moved to the tRNA gene cluster downstream of the A+T-rich region together with the other four tRNA genes, then it inverted and moved to the *nad1-trnL_1_* junction together with *nad2*. This is a more parsimonious scenario than independent movement with inversion of both genes (*trnE* and *nad2*) to the same gene junction. We also compared the mt genome organization of *Ceraphron* sp. with another ceraphronoid taxon, *Conostigmus* sp. (fig. 3), the sequence of which was reported in a previous study (Mao et al. 2014). In both ceraphronoids, the *trnQ*, *trnN*, *trnS_1_,* and *trnV* genes have moved to the tRNA gene cluster upstream of the *cox1* gene, whereas the *trnW* gene has inverted. The *nad2* gene has rearranged in both taxa but into different positions. The rearrangement of *nad2* has also been reported in two chalcidoid taxa, *Nasonia* and *Philotrypesis*, in which *nad2* has also moved into different positions (Oliveira et al. 2008; Xiao et al. 2011). The most striking gene rearrangement in both ceraphronoids is that the two rRNA genes are separated by protein-coding genes (*nad6* in *Ceraphron* sp. and *nad2* in *Conostigmus* sp.) instead of *trnV* lying between them, which has not been observed in other Hymenoptera. We consider that the existence of different protein-coding genes between the two rRNA genes might be useful markers to resolve the internal relationships of the Ceraphronoidea.

### The mt Genome of *Gasteruption* sp.

The *Gasteruption* sp. mt genome possesses 11 gene rearrangements (two protein-coding genes and nine tRNA genes) and a duplication of the A+T-rich region (fig. 4) relative to the ancestral hymenopteran. The *cox1* gene has inverted and moved into a position upstream of the A+T-rich region. The *nad2* gene has also inverted but remains in its ancestral location. Among the rearranged tRNA genes, most of them have inverted and moved to positions remote from their ancestral locations, although *trnY* has inverted, but remains in its ancestral location (downstream of *nad2*). In addition, *trnQ* has moved to a position downstream of *nad2*. The existence of duplicate A+T-rich regions separated by *trnL_2_* is consistent with the duplication/random loss model (Moritz et al. 1987). A parsimonious explanation is the *cox1* and *trnL_2_* genes inverted and moved to the position upstream of the A+T-rich region simultaneously (*cox1-trnL_2_*→*trnL_2_*-*cox1*), then a duplication of *cox1*, *trnL_2_* and the A+T-rich region followed by random deletions of the redundant copy of the *cox1* and *trnL_2_* genes occurred. An important evidence for this explanation is that two copies of a 10-bp complementary repeat exist at the beginning of *rrnS* and at the end of *trnL_2_* (AAAAGTATTT in *rrnS* and TTTTCATAAA in *trnL_2_*). We consider this short repeat may promote the *cox1* and *trnL_2_* inversion and long-range movement into the region between *rrnS* and A+T-rich region via recombination (Dowton and Campbell 2001; Mao et al. 2014). We then compared the mt genome organization of *Gasteruption* sp. with another two evanioid taxa, *Evania appendigaster* and *Pristaulacus compressus* (fig. 4). The gene positions are relatively conserved in *Evania appendigaster* and *Pristaulacus compressus* with only four and three gene rearrangements, respectively, when compared with the ancestral pancrustacean/hymenopteran. Although two tRNA genes (*trnS_2_* and *trnW*) have inverted in both *Gasteruption* sp. and *Evania appendigaster*, no mt gene rearrangements are shared among the three taxa. Similarly, *trnS_2_* is no longer adjacent to *cob* in all three taxa, but it has different, derived positions. Thus, among the three sequenced evanioid mt genomes, we identified 18 independent gene rearrangements, indicating that these could be extremely useful characters for assessing phylogeny in this superfamily.

### The mt Genomes of *Megalyra* sp. and *O. pulchella*

Figure 5 shows the mt genomes of *Megalyra* sp. and *O. pulchella* sequenced in this study. *Megalyra* sp. is from the superfamily Megalyroidea, whereas *O. pulchella* is from the superfamily Trigonalyoidea. The sister relationship of these two superfamilies has been recovered by some molecular analyses (including this study, see below) (Dowton et al. 1997; Dowton and Austin 2001; Castro and Dowton 2006; Heraty et al. 2011; Klopfstein et al. 2013), so we illustrate them in one figure to facilitate comparisons. Both of them possess dramatic gene rearrangements when compared with the ancestral pancrustacean/hymenopteran. In *Megalyra* sp., there are eight gene rearrangements. The most striking rearrangement is the one in which the two rRNA genes have inverted and moved into the *nad2-cox1* junction. Inverted rRNA genes have been reported in other insect orders, the Phthiraptera and Thysanoptera (Shao and Barker 2003; Covacin et al. 2006; Cameron et al. 2007, 2011). Interestingly, these four studies reported that both rRNA genes were inverted, similar to our observations in the *Megalyra* sp. mt genome. We suspect that there might be an advantage to having the two rRNA genes encoded on the same strand in insects. One candidate mechanism responsible for this phenomenon is the transcriptional feedback system regulating mtDNA replication. In this mechanism, the initiation of mtDNA replication is hypothesized to be positively correlated with the transcription rate, with the latter negatively regulated by the accumulation of gene products. Therefore, mtDNA replication is indirectly regulated by the amount of synthesized products (Axel and Thomas 2014). The rate of rRNA synthesis was found to be up to 60-fold higher than mRNA synthesis (i.e., of protein-coding genes) in humans due to the transcription termination factor binding mtDNA immediately downstream of the two rRNA genes (Martin et al. 2005). A significant difference between the transcription levels of rRNA and mRNA was also found in *Drosophila* (Torres et al. 2009). We suspect that the hymenopteran mt genome might also have a higher rate of rRNA synthesis. Therefore, the mechanism of transcriptional feedback system regulating mtDNA replication would confer an advantage to maintaining both rRNA genes on the same strand, as regulation of the synthesis of both rRNA genes would occur concomitantly. The retention of the rRNA genes on the minor strand in other available evaniomoph mt genomes indicates that the inversion occurred after the divergence of the Megalyroidea. In *O. pulchella*, 4 protein coding genes (*nad2*, *cox1*, *nad6,* and *cob*) and 10 tRNA genes have rearranged relative to the ancestral positions. *nad2* and *cox1* have moved into the position upstream of the A+T-rich region and the *nad3-nad5* junction, respectively. *nad6* and *cob* have inverted and swapped positions (*nad6-cob*→*cob*-*nad6*). The gene boundary *cob*-*nad6* was also characterized in *Venturia canescens* (Ichneumonoidea) and *Cotesia vestalis* (Ichneumonoidea) (Dowton, Cameron, Dowavic, et al. 2009; Wei, Shi, et al. 2010), similar to our observations in *O. pulchella*. However, these two superfamilies (Trigonalyoidea and Ichneumonoidea) are not closely related, indicating that this arrangement has likely evolved on two or three occasions independently. Therefore, it is probably one of the few examples of convergent evolution of gene order involving protein-coding gene rearrangements. Two tRNA genes (*trnQ* and *trnY*) have inverted in both *Megalyra* sp. and *O. pulchella*, but they are in different, derived positions. In *Megalyra* sp., both *trnQ* and *trnY* remain in their ancestral locations, whereas in *O. pulchella*, they have moved to the position downstream of *rrnS*. Furthermore, *trnH* is no longer between *nad5* and *nad4*, but the derived positions are different in the two taxa.

### Evolutionary Dynamics of Evaniomorph mt Genomes

A total of 64 gene rearrangements were identified in the seven evaniomorph mt genomes, including 4 local translocations (within a tRNA gene cluster and without inversion), 32 gene inversions, and 49 long-range movements (some genes were involved in two rearrangement events). The local translocations are readily explained by the duplication/random loss model (Moritz et al. 1987), whereas the dominant rearrangement events (inversion and long-range movement) are more consistent with the intramolecular recombination mechanism (Dowton and Campbell 2001; Mao et al. 2014). In this mechanism, the repeat fragments located around the breakpoints may play an important role in facilitating the recombination (Mao et al. 2014). The A+T-rich region (control region) has been suggested as a “hot spot” of recombination (Kurabayashi et al. 2008). Among the 64 gene rearrangements, there are 43 rearrangements that occurred close to the A+T-rich region. A series of repeat sequences detected in each evaniomorph mt genome A+T-rich region may have played a role in the gene rearrangements, via recombination (table 2).

**Table 2:** The Number of Repeats (Including Direct and Inverted Repeats) within the A+T-Rich Region and Gene Rearrangements Identified for Seven Evaniomorph Taxa

Species	12–20 bp	21–50 bp	51–100 bp	>100 bp	Close^a^	Not Close^a^	Inversion	Long Range	Local
*Ceraphron* sp.	10	8	0	0	11	1	5	10	0
*Conostigmus* sp.	23	0	9	2	5	7	1	11	1
*Evania appendigaster*	35	31	0	0	4	0	3	3	1
*Gasteruption* sp.	50	0	0	2	7	4	10	7	0
*Megalyra* sp.	84	31	0	4	5	3	6	6	0
*Pristaulacus compressus*	37	0	0	0	3	0	0	2	1
*Orthogonalys pulchella*	21	2	5	2	8	6	7	10	1

Note.—^a^Close/not close refers to whether the gene rearrangements occurred close to or far from the A+T-rich region. The definition of “close to the A+T-rich region” is 1) when a tRNA gene moves to or moves out from a position between the *rrnS-nad2* gene junction or 2) when a tRNA moves together with *rrnS* or *nad2*.

There have been few studies on the association of repeats with rearrangements in the mt genome. In a recent study on the plastid genome (Weng et al. 2014), repeats were strongly associated with the breakpoints in the rearranged genomes. In particular, large repeats (>20 bp and >60 bp) were significantly correlated with the degree of genome rearrangement. To better understand the relationship between repeats and gene rearrangements, we conducted a similar analysis for the A+T-rich region and the entire mt genome among the seven evaniomorph taxa. However, no significant correlations between the size of repeats and gene rearrangements could be detected (the number of different size of repeats in the A+T-rich region is listed in table 2). We suspect that this might be due to the high degree of divergence between the taxa studied here, with the number and type of repeats that are present at the time of rearrangement being obscured by subsequent evolution.

### The Utility of Gene Rearrangements for Assessing Phylogeny in the Evaniomorpha

Gene rearrangement characters have been suggested as reliable markers for deducing phylogenetic relationships (Boore and Brown 1998; Dowton et al. 2002). To explore the potential phylogenetic signals in the evaniomorph mt gene orders, such as shared, derived gene rearrangements, we visually inspected each pairwise comparison of gene orders in the output diagrams from CREx. A large number of individual gene rearrangements were detected between the evaniomorph mt genomes. However, no shared, derived gene rearrangements could be identified. This result indicates that the frequency of gene rearrangements might still be too high to be useful for assessing higher-level relationships. We investigate this point further in the following section.

### Rates of Gene Rearrangement in the Evaniomorpha

Does the Evaniomorpha have an intermediate rate of gene rearrangement? To answer this question, we calculated the breakpoint numbers of the evaniomorph, hemipteroid (hemipteran orders; Hemiptera, Phthiraptera, Psocodea, Thysanoptera), and nematoceran (Diptera: Nematocera; crane flies, gnats, and mosquitoes) mt genomes relative to the ancestral organization of insects. The hemipteroids (excluding the Hemiptera) have been identified as a group with a high rate of mt gene rearrangement (e.g., Shao et al. 2001, 2003), whereas most Nematocera have a low rate of mt gene rearrangement (e.g., Beckenbach 2012). As presented in table 3, the breakpoint numbers of the seven evaniomorph taxa range from 5 to 21, whereas the numbers of the three hemipteroid taxa (i.e., excluding *Triatoma* from the Hemiptera) are high (15–35), with most nematocerans low (0–5), although *Paracladura* is an obvious exception. These numbers confirm the intermediate rate of gene rearrangement of the Evaniomorpha. However, the inability to identify shared, derived gene rearrangements indicates that the rate of the gene rearrangement is still too high to be reliably used for reconstructing phylogenetic relationships. Therefore, increasing taxonomic sampling, particularly in those evaniomorph groups with a relatively lower rate of gene rearrangement might be more useful to establish phylogenetic relationships. Within the Evaniomorpha, the three evanioid taxa yield the lowest breakpoint numbers, which suggests the Evanioidea is the most promising candidate for further analyses. Although the monophyly of the Evanioidea was not recovered by some previous research (Dowton and Austin 2001; Castro and Dowton 2006), two most recently comprehensive studies robustly supported its monophyly (Heraty et al. 2011; Klopfstein et al. 2013).

**Table 3:** Breakpoint Numbers of the Evaniomorph, Hemipteroid, and Nematoceran mt Genomes Relative to the Ancestral mt Genome Organization

Species	Number of Breakpoints	Accession Number	References
Hemipteroids			
*Triatoma*	0	NC_002609	Dotson and Beard (2001)
*Heterodoxus*	35	NC_002651	Shao et al. (2001)
*Thrips*	30	AF335993	Shao and Barker (2003)
*Lepidoscopid*	15	AF335994	Shao et al. (2003)
Diptera: Nematocera			
*Anopheles*	5	NC_000875	Mitchell et al. (1993)
*Culicoides*	0	NC_009809	Matsumoto Y, Yanase T, Tshuda T, Noda H, unpublished
*Cramptonomyia*	0	JN861747	Beckenbach (2012)
*Tipula*	0	JN861743	Beckenbach (2012)
*Sylvicola*	0	JN861752	Beckenbach (2012)
*Paracladura*	21	JN861751	Beckenbach (2012)
*Arachnocampa*	2	JN861748	Beckenbach (2012)
*Mayetiola*	12	GQ387648	Beckenbach and Joy (2009)
Hymenoptera: Evaniomorpha			
*Megalyra*	16	KJ577600	Present study
*Gasteruption*	14	KJ619460	Present study
*Evania*	10	FJ593187	Wei, Tang, et al. (2010)
*Pristaulacus*	5	KF500406	Wei et al. (2013)
*Conostigmus*	16	KF015227	Mao et al. (2014)
*Ceraphron*	18	KJ570858	Present study
*Orthogonalys*	21	KJ619461	Present study

### Phylogenetic Analysis

Previous phylogenetic analyses of hymenopteran mt genome data by our group has established that uncontroversial relationships are only recovered with partitioned, model-based (Bayesian) analyses of nucleotide data (Dowton, Cameron, Austin, et al. 2009). Analysis of amino acid sequences using either maximum parsimony or Bayesian approaches failed to recover these uncontroversial relationships, as did analysis of nucleotide sequence data using the maximum parsimony criterion. Although our earlier studies also found that the exclusion of third codon positions markedly improved the recovery of these relationships, more recently we have found that the use of PartitionFinder (Lanfear et al. 2012) now facilitates the inclusion of third codon positions (Mao and Dowton 2014). PartitionFinder tends to group proteins into the strand on which they are encoded, an approach that we did not use in our earlier studies. For this reason, we analyzed the present data set using model-based analyses of nucleotide data, which was partitioned by PartitionFinder. Nevertheless, to assess how sensitive our phylogeny was to the analytical approach, we analyzed our data set using eight analytical approaches, which are outlined below.

**Fig. 6.—:**
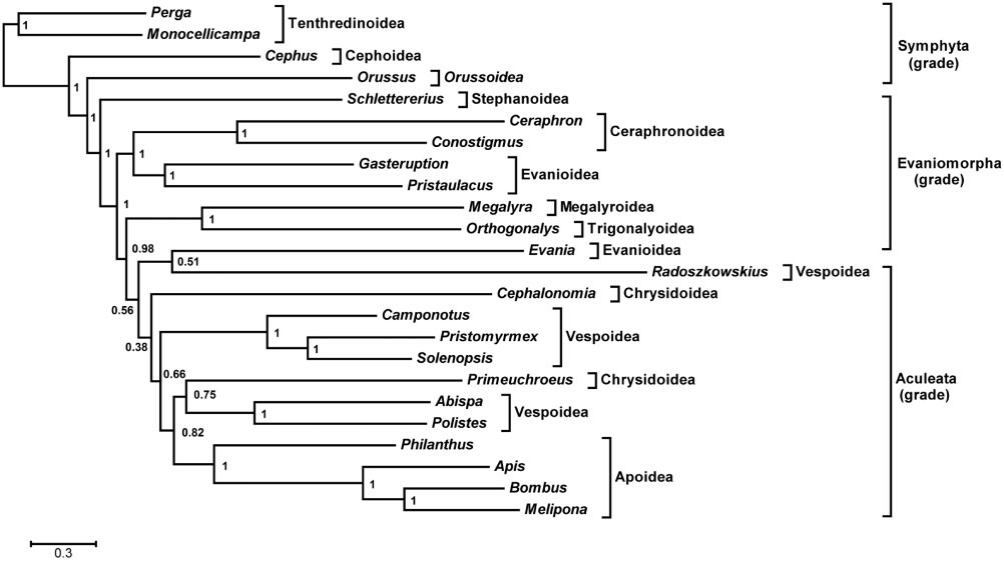
Bayesian analysis of the initial taxon data set (without rogue taxon deletion) based on mitogenomic sequences including 13 protein-coding genes and two rRNA genes. All the Symphyta, Evaniomorpha, and Aculeata were recovered as paraphyletic grades. Posterior probabilities are shown at each node.

We analyzed two taxon data sets (with and without rogue taxon deletion) using two alignment approaches (Muscle and MAFFT) and two phylogenetic approaches (maximum likelihood and Bayesian inference). Our initial data set contained all of the available evaniomorph and aculeate species. The trees yielded a number of expected relationships with high support (posterior probabilities 0.98–1.00 in Bayesian analyses and bootstrap proportions 57–100 in maximum likelihood analyses). For example, the Ceraphronoidea, Apoidea, Formicidae, and Vespidae were consistently recovered as monophyletic (fig. 6 and supplementary fig. S2, Supplementary Material online). However, some traditionally monophyletic groups failed to be recovered in all of the analyses. For example, the two chrysidoid genera (*Cephalonomia* and *Primeuchroeus*) failed to group together; *Evania* was placed within the Aculeata and grouped as a sister of *Radoszkowskius*. This latter relationship was also reported in a recent mt phylogeny of the Hymenoptera (Kaltenpoth et al. 2012). We then investigated whether some taxa were being placed in variable positions during the bootstrap and Bayesian analyses and as a result were obscuring other, more stable relationships (Wilkinson 1996). We prefer this objective approach to taxon pruning, rather than basing taxon exclusion upon, for example, inconsistency with previous phylogenetic hypotheses. Therefore, we conducted rogue taxon identification on all of the tree sets using RogueNaRok. For the RAxML bootstrap tree sets, *Evania appendigaster* and *Primeuchroeus* spp. were consistently identified as rogue taxa, whereas only *Primeuchroeus* spp. was identified in Bayesian tree sets. For consistency between analyses, we removed the concatenated sequences corresponding to the two rogue taxa (*Evania* and *Primeuchroeus*) and repeated the sequence alignment (as removal of rogue taxa might impact on the alignment) and phylogenetic analyses. The phylogenetic resolution was improved compared with the initial analyses. For example, a monophyletic Aculeata was consistently recovered in all analyses. Therefore, our discussion below focuses on the trees generated by the data set with these two taxa removed.

**Fig. 7.—:**
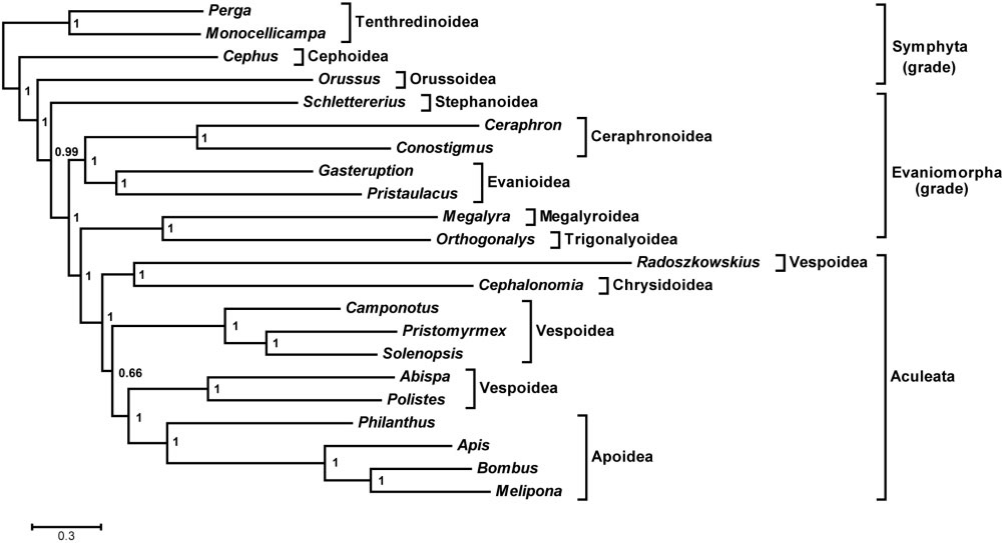
Bayesian analysis of the reduced taxon data set (with rogue taxon deletion) based on mitogenomic sequences including 13 protein-coding genes and two rRNA genes. Both the Symphyta and Evaniomorpha were recovered as paraphyletic grades. Posterior probabilities are shown at each node.

The general topologies were broadly congruent across the four analyses with similar levels of support for major clades (fig. 7 and supplementary fig. S3, Supplementary Material online). The two alignment approaches had no effect on topology and limited effect on nodal support (fig. 7 and supplementary fig. S3*A*, Supplementary Material online). When the two phylogenetic inference approaches are compared, there is only one topological difference, which was caused by the variable positions of *Radoszkowskius* (Vespoidea) and *Cephalonomia* (Chrysidoidea). In Bayesian analyses, the two taxa grouped together, and this clade was sister to Apoidea + the remaining Vespoidea (fig. 7 and supplementary fig. S3*A*, Supplementary Material online). However, in maximum likelihood analyses, *Radoszkowskius* was recovered as the sister group to the remaining Aculeata and *Cephalonomia* formed a clade with three formicid taxa (*Camponotus*, *Pristomyrmex,* and *Solenopsis*) (supplementary fig. S3*B* and *C*, Supplementary Material online). Nodal support was generally stronger for the Bayesian than the maximum likelihood analyses as has been commonly noted (Cameron et al. 2012; Nelson et al. 2012). The data matrix and the two Bayesian trees in figures 6 and 7 have been deposited in TreeBASE (accession number S16039, http://treebase.org/, last accessed June 30, 2014).

In the pruned data set, there are 22 hymenopteran taxa, with four taxa coming from the Symphyta. Our analyses strongly supported the Cephoidea (*Cephus*) as sister to Orussoidea + Apocrita, which is congruent with previously morphological studies (Vilhelmsen 2001; Vilhelmsen et al. 2010). The Orussoidea (*Orussus*) was recovered as sister to the Apocrita with high nodal support.

Within the Apocrita, the Aculeata was consistently recovered as monophyletic. Ignoring the unstable positions of *Radoszkowskius* and *Cephalonomia*, the internal relationships are highly congruent with our previous study (Mao et al. 2012). Compared with the previous study, there are two new representatives: *Camponotus* from the Vespoidea and *Philanthus* from the Apoidea. As expected, *Camponotus* formed a clade with another two vespoid taxa: *Pristomyrmex* and *Solenopsis*. All these three taxa are from the same family Formicidae. *Philanthus* was recovered at the base of the Apoidea.

The Evaniomorpha was recovered as paraphyletic, with the Aculeata nested within them. Of the representatives from the Evaniomorpha, the Stephanoidea was placed as the sister group to the remaining Evaniomorpha + Aculeata. The sister relationship of the Ceraphronoidea and Evanioidea was also highly supported in each analysis. The Trigonalyoidea was recovered as sister to the Megalyroidea, and this clade was sister to the Aculeata. A paraphyletic Evaniomorpha with respect to the Aculeata has commonly been recovered in previous molecular studies (Dowton and Austin 2001; Castro and Dowton 2006; Heraty et al. 2011; Sharkey et al. 2011; Klopfstein et al. 2013). However, the likely sister group to the Aculeata remains controversial. In our previous studies, the Aculeata were generally recovered among a clade with the Stephanoidea, Trigonalyoidea, and Megalyroidea but with weak support (Dowton and Austin 2001; Castro and Dowton 2006). A recent analysis of combined morphological and molecular data supported a sister group of Aculeata + Evanioidea, but this clade was not supported by the morphology-only tree or individual gene trees (Sharkey et al. 2011). Additionally, some recent molecular analyses recovered the same sister group to the Aculeata as in this study, but the results were sensitive to the alignment approach (Heraty et al. 2011; Klopfstein et al. 2013). In contrast, in this study the Aculeata + (Trigonalyoidea + Megalyroidea) clade was insensitive to variations in alignment approach and phylogenetic inference approach. We consider this clade to be the best supported hypothesis, based on current evidence. The phylogenetic relationships among evaniomorph superfamilies are also far from settled. Since Rasnitsyn (1988) placed the Stephanoidea within the Evaniomorpha, there has been no robust morphological or molecular evidence to support this affiliation. Instead, most subsequent analyses placed the Stephanoidea as the most basal apocritan lineage (Whitfield, 1992; Dowton and Austin 1994; Vilhelmsen et al. 2010; Heraty et al. 2011; Peters et al. 2011; Sharkey et al. 2011). Our results supported the basal position of the Stephanoidea but failed to give a clear indication to the affiliation of this superfamily. The sister relationship between the Trigonalyoidea and Megalyroidea has been consistently proposed in molecular analyses (Dowton et al. 1997; Dowton and Austin 2001; Castro and Dowton 2006; Heraty et al. 2011; Klopfstein et al. 2013). However, recent morphological analyses favor the Ceraphronoidea as the sister to the Megalyroidea based on mesosomal characters (Vilhelmsen et al. 2010). Our results confirmed the Trigonalyoidea + Megalyroidea clade with robust molecular support and yielded a novel sister relationship of the Ceraphronoidea and Evanioidea. The inclusion of other taxa is required to further test this novel relationship.

To date, most molecular phylogenies on the internal relationships of the Apocrita have relied on relatively short mt and nuclear gene fragments. The few studies that have used the much larger mt genome to assess this group have done so with relatively few taxa—usually one representative from each superfamily. Our study is the first attempt to use the entire mt genome, and a denser taxonomic sampling, to reconstruct phylogeny. Nevertheless, we do so for just two of the four apocritan groups, the Evaniomorpha and Aculeata. The molecular phylogenetic hypothesis presented here provides a reliable basis for future analyses.

## Supplementary Material

Supplementary figures S1–S3 are available at *Genome Biology and Evolution* online (http://www.gbe.oxfordjournals.org/).

Supplementary Data
