# Supplementary material for: Evolutionary Dynamics of the Mitochondrial Genome in the Evaniomorpha (Hymenoptera)—A Group with an Intermediate Rate of Gene Rearrangement
Source: Genome Biol Evol. 2014 Jul 3;6(7):1862–74. doi: 10.1093/gbe/evu145 (PMC4122943; doi:10.1093/gbe/evu145)
Supplement: Supplementary Data [file supp_6_7_1862__index.html]

Evolutionary dynamics of the mitochondrial genome in the Evaniomorpha (Hymenoptera) – a group with an intermediate rate of gene rearrangement — Evolutionary Dynamics of the Mitochondrial Genome in the Evaniomorpha (Hymenoptera)—A Group with an Intermediate Rate of Gene Rearrangement — Supplementary Data 

# Evolutionary Dynamics of the Mitochondrial Genome in the Evaniomorpha (Hymenoptera)—A Group with an Intermediate Rate of Gene Rearrangement

## Supplementary Data

files

**Files in this Data Supplement:**

- Supplementary Data - pdf file
- Supplementary Data - pdf file
- Supplementary Data - pdf file
