# Supplementary material for: Evolutionary Dynamics of the Mitochondrial Genome in the Evaniomorpha (Hymenoptera)—A Group with an Intermediate Rate of Gene Rearrangement
Source: Genome Biol Evol. 2014 Jul 3;6(7):1862–74. doi: 10.1093/gbe/evu145 (PMC4122943; doi:10.1093/gbe/evu145)
Supplement: Supplementary Data [file supp_evu145_supplementary_figure_1.pdf]

A

***Ceraphron* sp.**

NTR1: TAATTTTTACAATTATAT

NTR2: ATACCTCAAATTTAACATTTTTACCT

NTR3: ATTTAATTATATAAATATAGTATATA

***Gasteruption* sp.**

TR1: CTATTAATTAA

***Megalyra* sp.**

TR1: A(T)TAATGC(T)AGGATTTTCCCT(C)TAATAAAT

TR2: TTAATAAATATG(A)AGATTTATAAATCAATAGATTTATAAATCTTTTTAC(T)CC

**NTR1: AATTTAAAAAATTTCTTTCTATTG(A)AAGGGATAAAATCGTATTTTGTT(G)GTAG
AAATAAGAAACATGGGATAAATAGGGATTAACAAATAAATAAGTTATCAGGAAGGGGG**

**TR3: AATAATTTATTAATATAAAACTATAAATATACGGGAGTATATAGTAAAAAATCAATT
TATATAAATTTAAATTTAAATATATTTATTATTTAACTTTATTTAAAGATATTAATATTAAAACCTACTGA(G)AATTATGTTTACTATTAAGA
ATTAATTTATATGTAAGAAACGGATTTTTGAATAATTAAGAAAATAAATACCTAATCGCAG(A)GAGAAAATATAAAATGAATTAATTATATTA
ATAATAGTAAATTTCATTAATGAAATTAATCGTGATATTCGTTCGACAGATCTGATCAATAAGTAAATAACATTCACATATAAGAGAATTTCGT
TGTACTATTCTAATTAAAAAATAAATTAAGCATCTTGCTAAAATTT(A)AACAATAAAAAA**

TR4: C(T)AT(A)ATAAATATTTAATAAA

Orthogonalys pulchella

NTR1: TTTAATTTTATTAAATAATT

See part C for the DNA sequence of NTR2 and R1.

B

```
                   10        20        30        40        50        60        70        80
         ....|....|....|....|....|....|....|....|....|....|....|....|....|....|....|....|
19_AT1   TATTATTAATTTTCAATTATTAAATAAATATAATACTATAAATAATTATATATTATTAAATATATGATATTAAATTTAAA
19_AT2   -----------------------.........................................................

                   90       100       110       120       130       140       150       160
         ....|....|....|....|....|....|....|....|....|....|....|....|....|....|....|....|
19_AT1   ATGATTAATTATAATAAAGAAAAATATCAAATTTAACATATTAAAGAATTAAATTTTTATAAATGCAATTTTATAATATT
19_AT2   ................................................................................

                  170       180       190       200       210       220       230       240
         ....|....|....|....|....|....|....|....|....|....|....|....|....|....|....|....|
19_AT1   AAATTAAATTAATTTTTACAAAAAATTTTAATATAAAATTAAAAATTTATTTAATATAAATATTAAATTTTTTATATGAA
19_AT2   ................................................................................

                  250       260       270       280       290       300       310       320
         ....|....|....|....|....|....|....|....|....|....|....|....|....|....|....|....|
19_AT1   AATATTATAAAATAAAAATATTTTACACAAAAAGATGAAAATATGAATATTATATTAAATTTAATCAACCCAATAATTAT
19_AT2   ................................................................................

                  330       340       350       360       370       380       390       400
         ....|....|....|....|....|....|....|....|....|....|....|....|....|....|....|....|
19_AT1   AGTTGTCCTACGATTCTTTTTTTTTCTTTGGTTTCTGTAAAATTTATAATTAATGTCCATAAACCTGATGAAAATTAACC
19_AT2   ................................................................................

                  410       420       430       440       450       460       470       480
         ....|....|....|....|....|....|....|....|....|....|....|....|....|....|....|....|
19_AT1   TAATGTTCGATCATATAAAATTTTATATTTAAGTAGAACAAATTAACATAAAGTAAAATTTAATTAATGTTCGTTTTCGT
19_AT2   ................................................................................

                  490       500       510       520       530       540       550       560
         ....|....|....|....|....|....|....|....|....|....|....|....|....|....|....|....|
19_AT1   ATAAAAGCTATATTTTATATAAGAATAATGATGTATATAAATTAAGATAATTATACATAATATTATGATTGAAAATTTTA
19_AT2   ................................................................................

                  570       580       590       600       610       620       630       640
         ....|....|....|....|....|....|....|....|....|....|....|....|....|....|....|....|
19_AT1   GTGTAACTATTTAACATAATGTAATCTAAATTTATTATATTAAAATAAATTTTTTATAATATTAAATTAAATTTTGATGT
19_AT2   ................................................................................

                  650       660       670       680       690       700       710       720
         ....|....|....|....|....|....|....|....|....|....|....|....|....|....|....|....|
19_AT1   TAAATTATTTTTATAATGTATATATTCACTAAAAAATTAAAATTCTAAAAATCATAAAATAAATAATTATTTTATTTATT
19_AT2   ................................................................................

                  730       740       750       760       770       780       790       800
         ....|....|....|....|....|....|....|....|....|....|....|....|....|....|....|....|
19_AT1   TATATAAAAAAATTAAAATCAATCTATTAATTATTAATTAACTATTAATTAACTATTAATTAACTATTAATTAACTATTA
19_AT2   ................................................................................

                  810       820       830       840       850       860       870       880
         ....|....|....|....|....|....|....|....|....|....|....|....|....|....|....|....|
19_AT1   ATTAACTATTAATTAACTATTAATTAACTATTAATTAACTATTAATTAACTATTAATTAACTATTAATTAACTATTAATT
19_AT2   ..........................------------------------------------------------------

                  890       900       910       920       930       940       950       960
         ....|....|....|....|....|....|....|....|....|....|....|....|....|....|....|....|
19_AT1   AACTATTAATTAACTATTAATTAACTATTAATTAACTATTAATTAACTATTAATTAACTATTAATTAACTATTAATTAAC
19_AT2   --------------------------------------------------------------------------------

                  970       980       990      1000      1010      1020      1030
         ....|....|....|....|....|....|....|....|....|....|....|....|....|....|...
19_AT1   TATTAATTAACTATTAATTAACTATTAATTAACTATTAATTAACTATTAATTAACTATTAATTAACTATTAAT
19_AT2   -------------------------------------------------------------------------
```

C

```
                  10        20        30        40        50        60        70        80
         ....|....|....|....|....|....|....|....|....|....|....|....|....|....|....|....|
16 repeat1  TAAAAATTGAGATTATATATCCAAGTAAAAAATAAAATAGTTTAATTGGGGGGACAACTATTTGGTATATATTTACATAC
16 repeat2  ..................C.....A..............A........................................

                  90       100       110       120       130       140       150       160
         ....|....|....|....|....|....|....|....|....|....|....|....|....|....|....|....|
16 repeat1  TAAAAATTGAGATTATATATTCAGATTTGAATTTTTTTAATTAGAGGATAAATTTTATTCTATTTAAATTTAAAAATTTT
16 repeat2  ................................................................................

                 170       180       190       200       210       220       230       240
         ....|....|....|....|....|....|....|....|....|....|....|....|....|....|....|....|
16 repeat1  AAAAAAAGTGATAATTTTTTCTATAACAATTAGAATGAAATCGTAAATTATATTGTTAAATTTTTAAAGTTTATATATAT
16 repeat2  ................................................................................

                 250       260       270       280       290       300       310       320
         ....|....|....|....|....|....|....|....|....|....|....|....|....|....|....|....|
16 repeat1  TTATCTTTATAAGTATTTAATATATTATATTTATTAATCTATAAATTAATTTAACTTTTTATTAATCTTTAAATTAAAAT
16 repeat2  ................................................................................

                 330       340       350       360       370       380       390       400
         ....|....|....|....|....|....|....|....|....|....|....|....|....|....|....|....|
16 repeat1  AACTTTTTAAAATTAATATACTAATAAAATTATATTTTAAAAAATTATAAATAAATAATATAAAAACTTATAATATATTA
16 repeat2  ................................................................................

                 410       420       430       440       450
         ....|....|....|....|....|....|....|....|....|....|.
16 repeat1  TAAAGTAAATTATTTTATTTTATATAATAAATTTATTATTAATAATAAGTA
16 repeat2  ...................................................
```

Supplementary figure 1 A. The DNA sequence of different repeats identified in the A+T rich region of each evaniomorph mt genome. The repeat names are as in Fig. 2. The site difference among different copies is shown in the right brackets next to the variable site. B. DNA sequence alignment of the duplicated A+T rich regions in the *Gasteruption* sp. mt genome. C. DNA sequence alignment of the major portion of the A+T rich region in the *Orthogonalys pulchella* mt genome.
