# Supplementary material for: Evolutionary Dynamics of the Mitochondrial Genome in the Evaniomorpha (Hymenoptera)—A Group with an Intermediate Rate of Gene Rearrangement
Source: Genome Biol Evol. 2014 Jul 3;6(7):1862–74. doi: 10.1093/gbe/evu145 (PMC4122943; doi:10.1093/gbe/evu145)
Supplement: Supplementary Data [file supp_evu145_supplementary_figure_2.pdf]

A

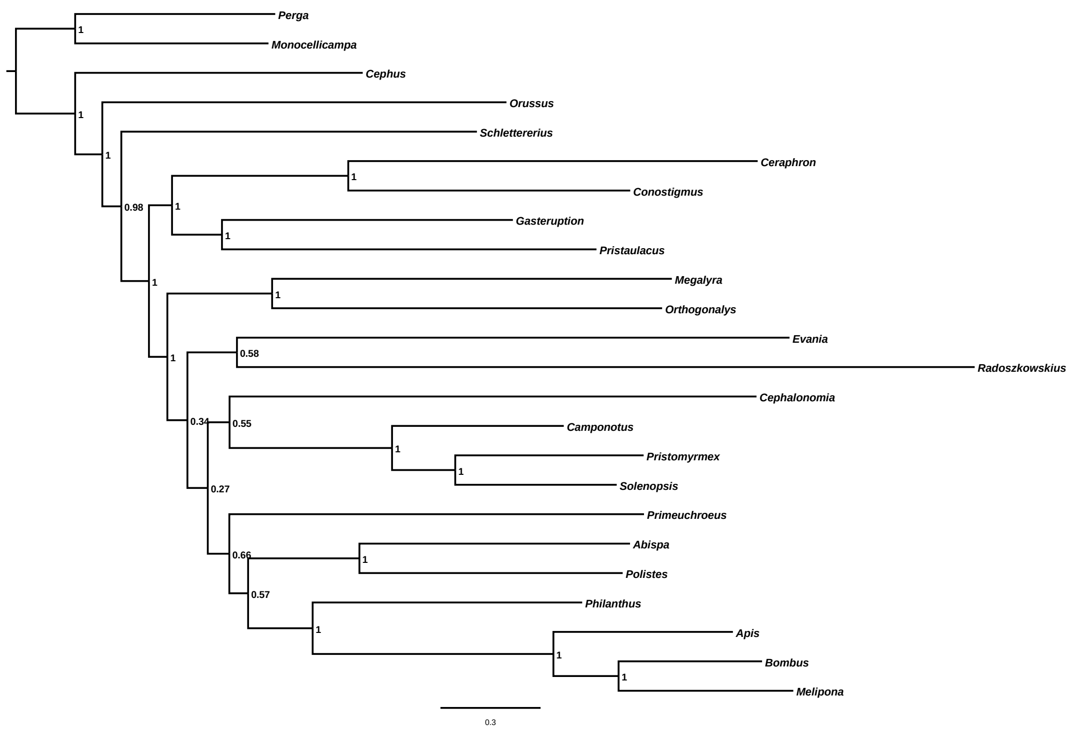
Perga
Monocellicampa
Cephus
Orussus
Schlettererius
Ceraphron
Conostigmus
Gasteruption
Pristaulacus
Megalyra
Orthogonalys
Evania
Radoszkowskius
Cephalonomia
Camponotus
Pristomyrmex
Solenopsis
Primeuchroeus
Abispa
Polistes
Philanthus
Apis
Bombus
Melipona
0.3

B

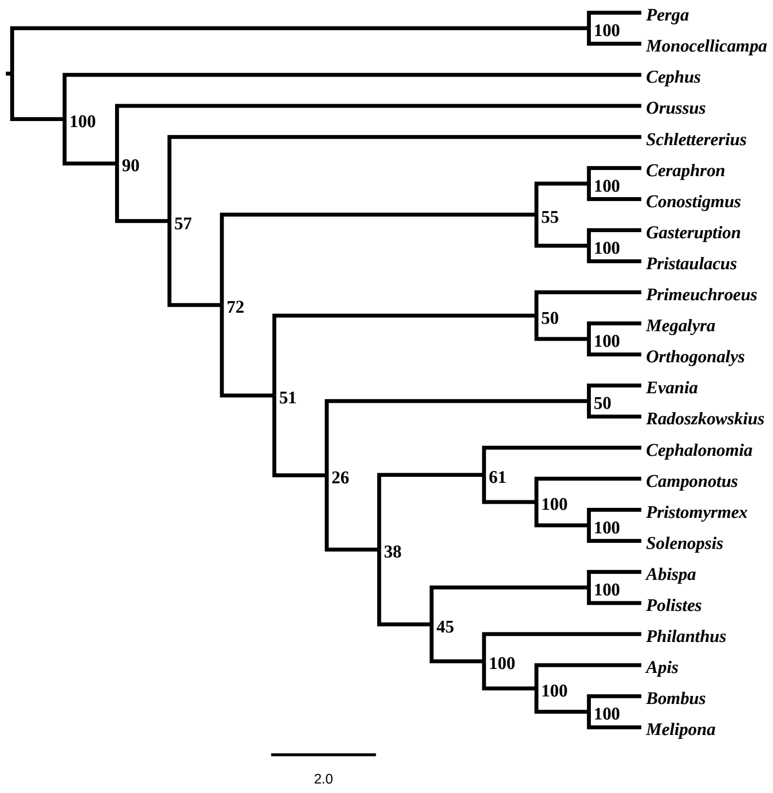
Perga
Monocellicampa
Cephus
Orussus
Schlettererius
Ceraphron
Conostigmus
Gasteruption
Pristaulacus
Primeuchroeus
Megalyra
Orthogonalys
Evania
Radoszkowskius
Cephalonomia
Camponotus
Pristomyrmex
Solenopsis
Abispa
Polistes
Philanthus
Apis
Bombus
Melipona
2.0

C

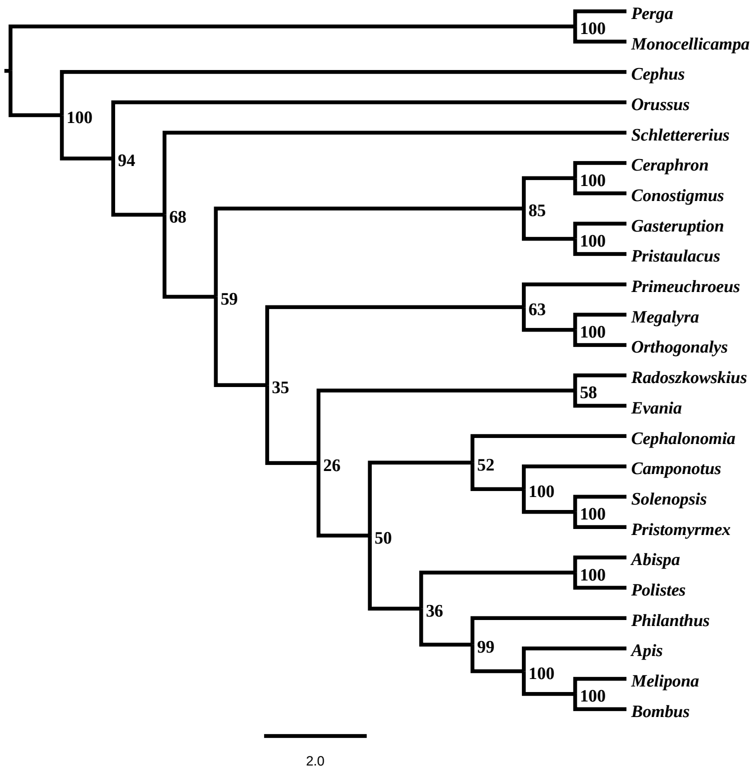

Supplementary figure 2. Phylogenetic trees of the initial taxon dataset (without rogue taxon deletion) based on mitogenomic sequences including 13 protein-coding genes, and two rRNA genes. A. Bayesian analysis of the MAFFT alignment dataset. Posterior probabilities are shown at each node. B. Maximum likelihood analysis of the MAFFT alignment dataset. Bootstrap percentages are shown at each node. C. Maximum likelihood analysis of the Muscle alignment dataset. Bootstrap percentages are shown at each node.
