# Supplementary material for: Evolutionary Dynamics of the Mitochondrial Genome in the Evaniomorpha (Hymenoptera)—A Group with an Intermediate Rate of Gene Rearrangement
Source: Genome Biol Evol. 2014 Jul 3;6(7):1862–74. doi: 10.1093/gbe/evu145 (PMC4122943; doi:10.1093/gbe/evu145)
Supplement: Supplementary Data [file supp_evu145_supplementary_figure_3.pdf]

A

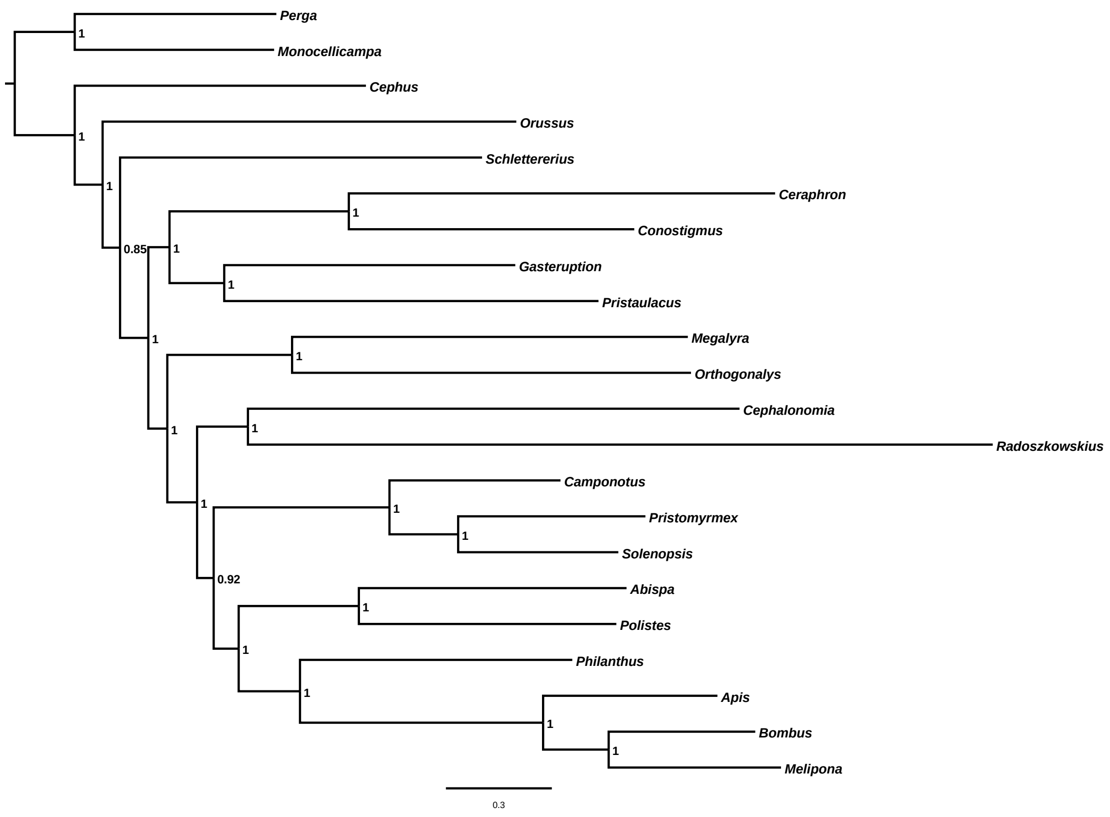

Perga
Monocellicampa
Cephus
Orussus
Schlettererius
Ceraphron
Conostigmus
Gasteruption
Pristaulacus
Megalyra
Orthogonalys
Cephalonomia
Radoszkowskius
Camponotus
Pristomyrmex
Solenopsis
Abispa
Polistes
Philanthus
Apis
Bombus
Melipona
0.85
0.92
0.3

B

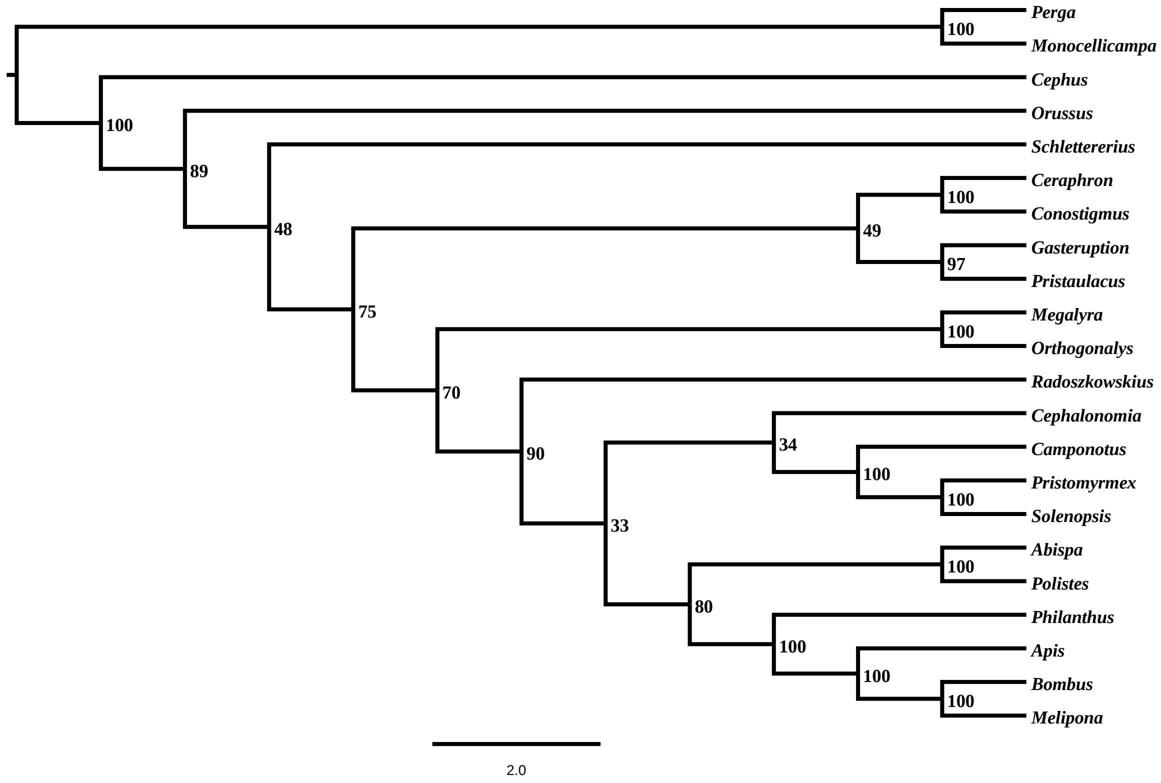

Perga
Monocellicampa
Cephus
Orussus
Schlettererius
Ceraphron
Conostigmus
Gasteruption
Pristaulacus
Megalyra
Orthogonalys
Radoszkowskius
Cephalonomia
Camponotus
Pristomyrmex
Solenopsis
Abispa
Polistes
Philanthus
Apis
Bombus
Melipona
100
89
48
75
70
90
33
80
49
97
34
2.0

C

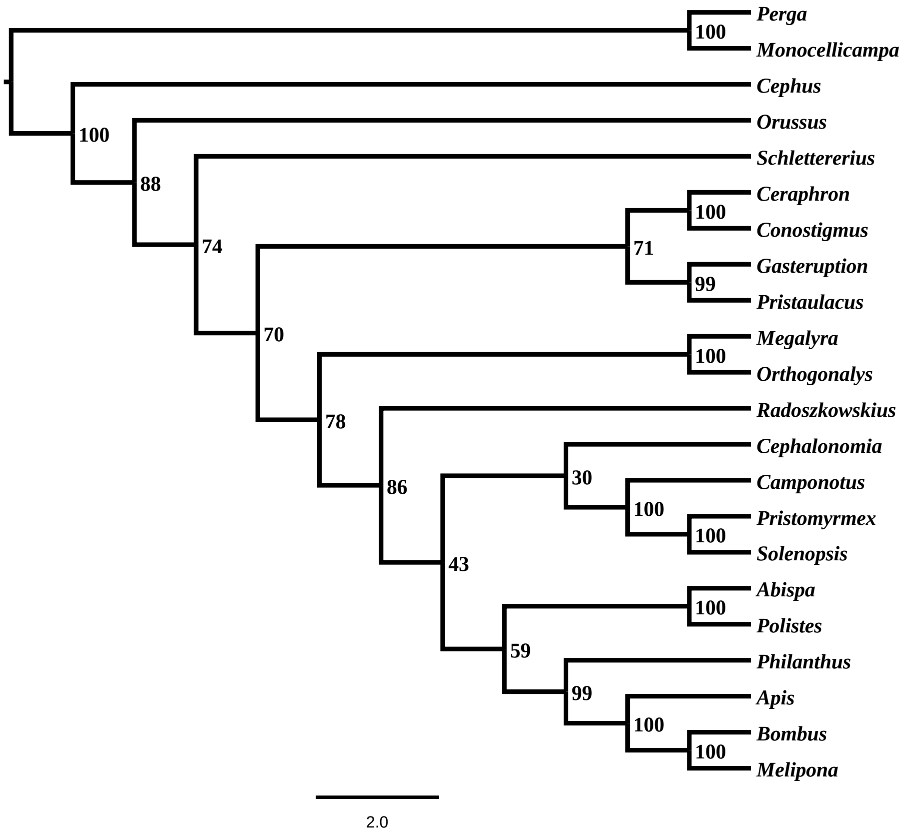

Supplementary figure 3. Phylogenetic trees of the reduced taxon dataset (with rogue taxa deletion) based on mitogenomic sequences including 13 protein-coding genes, and two rRNA genes. A. Bayesian analysis of the MAFFT alignment dataset. Posterior probabilities are shown at each node. B. Maximum likelihood analysis of the MAFFT alignment dataset. Bootstrap percentages are shown at each node. C. Maximum likelihood analysis of the Muscle alignment dataset. Bootstrap percentages are shown at each node.
